# The Effect of Lingual Resistance Training Interventions on Adult Swallow Function: A Systematic Review

**DOI:** 10.1007/s00455-019-10066-1

**Published:** 2019-10-14

**Authors:** Sana Smaoui, Amy Langridge, Catriona M. Steele

**Affiliations:** 1 Swallowing Rehabilitation Research Laboratory, KITE - Toronto Rehabilitation Institute - University Health Network, 550 University Avenue, 12th Floor, Toronto, ON M5G 2A2 Canada; 2 Rehabilitation Sciences Institute, Faculty of Medicine, University of Toronto, Toronto, M5G 1V7 Canada

**Keywords:** Deglutition, Deglutition disorders, Rehabilitation, Dysphagia, Swallowing, Tongue

## Abstract

Lingual resistance training has been proposed as an intervention to improve decreased tongue pressure strength and endurance in patients with dysphagia. However, little is known about the impact of lingual resistance training on swallow physiology. This systematic review scrutinizes the available evidence regarding the effects of lingual resistance training on swallowing function in studies using Videofluoroscopic Swallowing Studies (VFSS) with adults. Seven articles met the inclusion criteria and underwent detailed review for study quality, data extraction, and planned meta-analysis. Included studies applied this intervention to a stroke and brain injury patient populations or to healthy participants, applied different training protocols, and used a number of outcome measures, making it difficult to generalize results. Lingual resistance training protocols included anterior and posterior tongue strengthening, accuracy training, and effortful press against hard palate with varying treatment durations. VFSS protocols typically included a thin barium stimulus along with one other consistency to evaluate the effects of the intervention. Swallowing measures included swallow safety, efficiency, and temporal measures. Temporal measures significantly improved in one study, while safety improvements showed mixed results across studies. Reported improvements in swallowing efficiency were limited to reductions in thin liquid barium residue in two studies. Overall, the evidence regarding the impact of lingual resistance training for dysphagia is mixed. Meta-analysis was not possible due to differences in methods and outcome measurements across studies. Reporting all aspects of training and details regarding VFSS protocols is crucial for the reproducibility of these interventions. Future investigations should focus on completing robust analyses of swallowing kinematics and function following tongue pressure training to determine efficacy for swallowing function.

**Electronic supplementary material:**

The online version of this article (10.1007/s00455-019-10066-1) contains supplementary material, which is available to authorized users.

## Introduction

Lingual resistance training has emerged as an intervention for the rehabilitation of swallowing impairment, based on the fact that reduced tongue pressures have been found in adults with neurogenic dysphagia [1–3]. A recent systematic review by McKenna et al. [4] found converging evidence that gains in tongue strength can be expected after a course of isometric lingual strength training, but concluded that it remains unclear whether these strength gains generalize to improvements in swallow function. The intent of this systematic review is to look deeper into reported changes in swallowing function following lingual resistance training. We were specifically interested to scrutinize research using videofluoroscopic swallowing studies (VFSS) to measure changes in swallowing function, to evaluate and methodologically compare the VFSS protocols that have been used, and, if possible, to synthesize results across studies.

The tongue plays an important role in swallow function as it is composed of an intricate muscle structure allowing for fast and flexible posturing during oral functions [5–7]. During swallowing, its intrinsic and extrinsic muscles function synergistically to aid in bolus containment, loading, and the generation of a driving force exerted on the bolus to propel and squeeze it through the oropharynx [8–14]. Tongue strength has been investigated in a number of patient populations, including Parkinson disease [15–17], head and neck cancer [18–21], oculopharyngeal muscular dystrophy [22, 23], acquired brain injury [2], and cerebrovascular accident [1, 3]. Acute neurological impairments, such as stroke and brain injury, along with other progressive impairments, such as Parkinson disease, are known to be associated with high rates of swallowing impairment or oropharyngeal dysphagia [24]. In these patient populations, the tongue may fail to contain the bolus in the mouth or generate the necessary force to propel the bolus into the pharynx in a coordinated and controlled manner. Potential functional consequences of tongue weakness include impairments in swallow timeliness and airway closure resulting in penetration and/or aspiration (safety concerns) and the accumulation of residual material in the oropharyngeal cavities (efficiency concerns) [25].

Recent research has shown promising results for tongue strengthening exercises in building tongue strength and endurance in both healthy and disordered populations [1, 26–30], which has led to the increasing uptake of lingual resistance training protocols in swallowing rehabilitation [31]. However, whether improvements in swallowing function occur remains less clear. We were interested in further scrutinizing the available evidence regarding the effects of lingual resistance training on swallowing function. Our research questions were:

1. Which lingual resistance training protocols have been used to target improved swallowing function in adults?
2. How have changes in swallowing function been measured in VFSS?
  - Which stimuli have been used?
  - Which measures of swallowing biomechanics have been reported?
  - Which measures of swallowing safety have been reported?
  - Which measures of swallowing efficiency have been reported?
3. What other measures have been used to capture the impact of lingual resistance training protocols on swallowing?
4. What are the reported results of lingual resistance training protocols on
  - Tongue pressure generation?
  - Swallowing outcomes (biomechanics, safety, efficiency)?

## Methods

The Preferred Reporting Items for Systematic Reviews and Meta-Analyses (PRISMA) statement [32] was used to guide development and methodology of this systematic review.

### Search Strategy

An information specialist assisted in conducting a systematic search of the literature in AMED (Allied and Complimentary Medicine), Cochrane Central Register of Controlled Trials (CENTRAL), Embase, Ovid MEDLINE(R) (including Epub Ahead of Print, In-Process & Other Non-Indexed Citations, Ovid MEDLINE(R) Daily), CINAHL, and SpeechBite. Searches were conducted in each database from inception of the database until June 2018. Search strategies included the use of text words and subject headings (e.g. MeSH, Emtree) related to (1) *the tongue* (tongue, lingua*), (2) *swallowing* (swallow*, deglut*, dysphagi*, fluoro*, cinefluoro*, videofluoro*), and (3) *exercise training* (strength*, pressur*, exercise*, protocol*, intervention, train or training, rehab*, treatment). The use of an asterisk allowed searching for terms as root words or truncated terms and the return of all words containing that root word. Searches were limited to peer-reviewed studies published on human adults in English. The electronic search strategy for Ovid MEDLINE(R) (including Epub Ahead of Print, In-Process & Other Non-Indexed Citations, Ovid MEDLINE(R) Daily) can be found in Online Appendix A.

### Eligibility Criteria

Studies were included if they met the following criteria: (1) studied human subjects over the age of 18 years, (2) provided a tongue pressure intervention, (3) completed a baseline VFSS to outline swallow function pre-intervention, (4) performed a post-intervention VFSS to measure intervention effects on swallowing, (5) had abstract and full-text available in English, and (6) were published in a peer-reviewed journal. The review was restricted to randomized controlled trials, controlled studies, case–control studies, cohort studies, and case series designs. Single case studies were excluded from this review as we aimed to examine articles using representative samples of reasonable size. Studies characterizing tongue pressure without delivery of any intervention targeting the tongue were excluded. Additionally, studies validating new technologies or devices for measuring tongue pressure were also excluded as the aim of this review was to determine the effects of intervention and not on technological development.

Tutorials, educational reports, literature reviews, systematic reviews, book chapters, and conference abstracts were excluded because of their lack of prospective intervention design. Studies with pediatric populations and animals were also excluded from this review because our purpose was to investigate effects of lingual resistance training on the swallowing function of adults. We excluded studies with populations who had received surgical interventions to the head and neck, as we were interested in outcomes on functions of unaltered muscular anatomy. Similarly, studies that included patients who had received chemotherapy or radiation were excluded from this review as these treatments may further exacerbate swallowing impairment due to side effects including muscle fibrosis and neuropathy.

### Study Selection

**Fig. 1:**
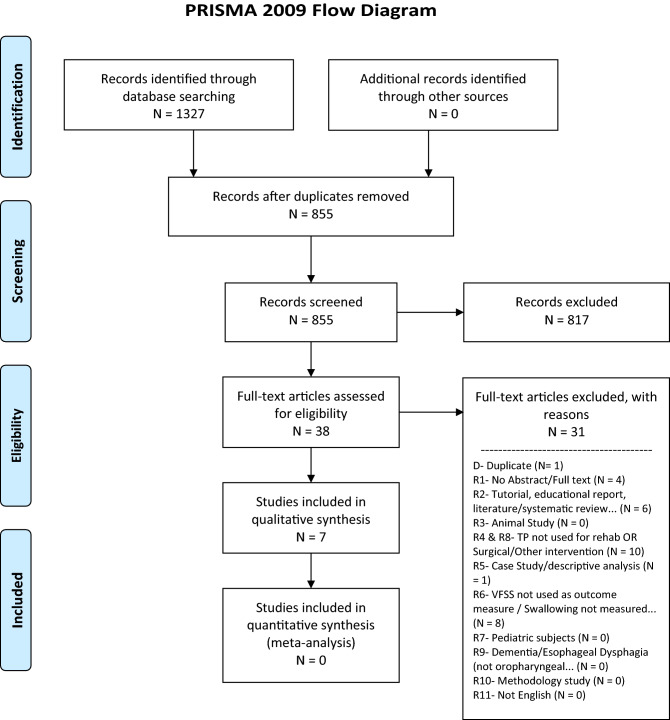
A PRISMA flow diagram depicting the different phases of the systematic review, mapping out the number of records identified, included and excluded

As shown in Fig. 1, the original search yielded a total of 1327 records, of which 472 were duplicates. After removal of these duplicates, two reviewers independently assessed the titles and abstracts of all retrieved records and determined their eligibility for potential inclusion. Cohen’s Kappa and percentage of inter-rater agreement were calculated to evaluate the level of agreement between both raters [33]. When ratings were conflicting, the article was retained for full-text review. Full-text articles of both accepted and conflicting ratings were then reviewed independently by both reviewers in order to determine whether the article should be included in the systematic review. If an article was not selected, a reason for exclusion was documented based on eleven rejection criteria (see Fig. 1). Disagreements in final ratings of full-text articles were resolved by consensus between both raters.

### Risk of Bias (Quality) Assessment

A methodological quality assessment of individual studies was completed independently by each reviewer to evaluate the validity of study design and reporting methods. Risk of bias evaluation was completed using the Cochrane Collaboration’s *Tool for Assessing Risk of Bias* [34]. The criteria assessed were selection bias (random sequence generation, allocation concealment), performance bias (blinding of participants and personnel), detection bias (blinding of outcome assessment), attrition bias (incomplete outcome data), and reporting bias (selective reporting). It was of particular interest to document whether a study included sufficient detail to permit replication when describing the intervention protocol, the VFSS procedures used (e.g., frame rate, stimuli used, number of trials), or the methods of VFSS analysis used (i.e., duplication of VFSS rating, and the use of valid and reliable operational definitions and assessment tools for VFSS analysis). Each item on the Cochrane Collaboration’s *Tool for Assessing Risk of Bias* was scored with a “Y” for yes if susceptible to bias in that category, “N” for no if not susceptible to bias in that category, and “U” for unsure/other if raters could not determine appropriate scores, if the criteria were not applicable, or if this was not reported for that particular category.

Quality in reporting was also scored using the NIH *Quality Assessment Tool for Before-After (Pre–Post) Studies with No Control Group* [35] to evaluate the internal validity of eligible studies with quasi-experimental, pre–post-intervention designs: (1) Studies that had the least risk of bias were classified as “good”, (2) those susceptible to bias were considered “fair” if this bias was not sufficient to invalidate their results, and (3) those as “poor” if they had a significant risk of bias. In cases where there was a disagreement in ratings, reviewers met and discussed their ratings until they achieved consensus.

### Data Extraction Process

Data extraction was completed independently by a single rater for full articles that met all inclusion criteria outlined above. A form was developed to standardize and capture the relevant data from each article (Online Appendix B). Data extraction included the following: (1) study design; (2) patient population descriptions (age, sex, etiology); (3) sample size; (4) proportion of males and females; (5) use of matched controls; (6) intervention details; (7) tongue strengthening device used—if applicable; (8) tongue intervention protocol (repetitions, frequency, duration); (9) VFSS protocol (stimuli, volumes and trials, frames/s); (10) outcome measure: swallow safety; (11) outcome measure: swallow efficiency; and (12) other visuo-perceptual or temporal swallowing parameters measured on VFSS. Results from each study including statistical analyses of changes in swallowing function after intervention were also extracted.

## Results

### Literature Retrieval

Figure 1 provides an overview of the selection process for included studies. Of the 855 studies identified for preliminary screening of titles and abstracts, 817 were rejected after failing to meet inclusion criteria. At abstract screening, the inter-rater agreement was 96.5% with a Cohen’s Kappa statistic of 0.41. Although high percent agreement was achieved, only moderate inter-rater agreement at the abstract screening level was suggested by the Cohen’s Kappa result, due to one rater’s tendency to rate items as unsure [36]. When examining ratings at the full-text level, levels of inter-rater agreement were 94.7% with a Cohen’s Kappa of 0.86 indicating almost perfect agreement between raters [36].

The most common reason for article exclusion (*N* = 487) was that tongue measurements were not collected pre- and post-interventions involving tongue-specific exercises, rather, tongue measurements were obtained to characterize impairment at a single time point, for other interventions not focused on the tongue, or to guide the development of new tools/technologies (e.g. video-ultrasonography, GlideScope®, algorithms and neural networks). Another common reason for exclusion was use of the single case study design (*N* = 105), regardless of whether tongue pressure training was utilized. Ultimately, a total of seven articles met the inclusion criteria for review and data extraction.

### Quality Assessment

Table 1 summarizes the quality assessment of all included studies using the Cochrane Risk of Bias Tool. Selection bias was found in four studies, where participants were enrolled either via convenience sampling or by consecutive recruitment without randomization. Quality assessment highlighted a high degree of performance bias in all studies included, where blinding of study participants to their allocated intervention group (if applicable) and of the personnel performing the intervention was not reported. Of the seven studies reviewed, two reported that they included some level of blinding of outcome assessor, where the clinicians rating the VFSS were blinded to participant [2, 3]. Detection bias was present in the remaining five studies, as there was no mention of blinding of the raters in the study for any outcome measures. Attrition bias was relatively low, with all participants completing the full intervention in four [1, 2, 29, 37] of the seven studies reviewed. Two studies [3, 38] were deemed to have a high risk of attrition bias as more than 20% of their participants were lost to follow-up. One study [39] did not report final sample size, and therefore it was not possible to determine whether any participants were lost to follow-up. Finally, reporting bias was deemed to be high in three studies [37–39] that did not provide any information regarding the stimuli used to assess swallowing function on VFSS.

**Table 1:** Cochrane tool for risk of bias

Study	Selection bias	Performance bias	Detection bias	Attrition bias	Reporting bias
Cho et al. [39]	+	+	+	?	+
Kim et al. [37]	−	+	+	−	+
Park et al. [38]	−	+	+	+	+
Robbins et al. [1]	+	+	+	−	−
Robbins et al. [28]	+	+	+	−	−
Steele et al. [2]	+	+	−	−	−
Steele et al. [3]	−	+	−	+	−

+  Yes to susceptibility of bias;   − not susceptible to bias; ?  unsure/could not determine appropriate rating

The quality assessment completed using the NIH tool deemed four studies to be “poor” in quality, two as “fair”, and one study as “good”(see Table 2). Four main reasons for low quality were found: (1) small sample sizes [1, 2, 29, 39]; (2) lack of clarity with regards to the intervention/service provided and whether this was delivered consistently to all patients [1, 29, 37, 39]; (3) use of valid and reliable outcome measures [2, 3, 37-39]; and (4) blinding of those providing the intervention or analyzing the data [1, 29, 37-39].

**Table 2:** NIH rating of bias using the quality assessment tool for before–after (pre–post) studies with no control group

Study		Risk of bias											
Author	Year	Was the study question or objective clearly stated?	Were eligibility/selection criteria for the study population prespecified and clearly described?	Were the participants in the study representative of those who would be eligible for the test/service /intervention in the general or clinical population of interest?	Were all eligible participants that met the prespecified entry criteria enrolled?	Was the sample size sufficiently large to provide confidence in the findings?	Was the test/service/intervention clearly described and delivered consistently across the study population?	Were the outcome measures prespecified, clearly defined, valid, reliable, and assessed consistently across all study participants?	Were the people assessing the outcomes blinded to the participants' exposures/interventions?	Was the loss to follow-up after baseline 20% or less? Were those lost to follow-up accounted for in the analysis?	Did the statistical methods examine changes in outcome measures from before to after the intervention? Were statistical tests done that provided p values for the pre-to-post changes?	Were outcome measures of interest taken multiple times before the intervention and multiple times after the intervention (i.e., did they use an interrupted time-series design)?	Quality rating (good, fair, poor)
Cho et al. [39]	2017	Yes	Yes	Yes	NR	No	No	CD	NR	CD	Yes	No	Poor
Kim et al. [37]	2017	Yes	Yes	Yes	Yes	Yes	CD	CD	NR	Yes	Yes	No	Fair
Park et al. [38]	2015	Yes	Yes	Yes	No	Yes	Yes	CD	NR	No	Yes	No	Poor
Robbins et al. [1]	2007	Yes	Yes	Yes	Yes	No	CD	Yes	NR	Yes	Yes	No	Fair
Robbins et al. [28]	2005	Yes	Yes	No	Yes	No	CD	Yes	NR	Yes	Yes	No	Poor
Steele et al. [2]	2013	Yes	NR	Yes	Yes	No	Yes	No	Yes	Yes	Yes	No	Poor
Steele et al. [3]	2016	Yes	Yes	Yes	Yes	Yes	Yes	CD	Yes	No	Yes	No	Good

*CD* cannot determine, *NR* not reported, *NA* not applicable

### Patient Characteristics

Patient characteristics can be found in Table 3. Three different patient population groups were included: stroke [1, 3, 37-39], acquired brain injury [2], and healthy participants [29]. Sample sizes varied widely across studies, ranging from six participants [2] to 29 participants [38]. Studies included both male and female participants; however, most studies included a larger proportion of males compared to females. The ages of participants enrolled across studies ranged from 32 [2] to 90 years [1]. Only three articles [3, 37, 38] included a group for comparison; however, the comparison group received some form of dysphagia intervention termed *conventional or traditional* exercise or an alternative tongue intervention protocol in all three cases.

**Table 3:** Patient characteristics

Study	Year	*N* (M, F)	Average age in years (Range)	Control Group	Etiology
Cho et al. [39]	2017	9 (NR)	NR	No	Stroke
Kim et al. [37]	2017	18 (11,7)	62.17 (51.16–73.18)	*N* = 17	Stroke
Park et al. [38]	2015	15 (6, 9)	67.3 (56.7–77.9)	*N* = 14	Stroke
Robbins et al. [1]	2007	10 (5, 5)	69.7 (51–90)	No	Stroke
Robbins et al. [28]	2005	10 (4, 6)	NR (70–89)	No	Healthy
Steele et al. [2]	2013	6 (4, 2)	42.33 (NR)	No	Acquired brain injury following motor vehicle accident
Steele et al. [3]	2016	5 (NR)	71 (49–89)	Tongue pressure profile Training group; * N* = 6	Stroke

*N *sample size, *M* male, *F* female, *NR* not reported

#### Question 1: Training Protocols

Tongue exercises included anterior and posterior tongue strengthening, tongue pressure accuracy training, and oral motor exercises of the tongue including effortful press against hard palate. The Iowa Oral Performance Instrument (IOPI) was the primary device used for lingual resistance training across the studies identified [1-3, 29, 37, 38]; however, one study used no tool at all [39]. A large variation was also found in the treatment durations including 4, 5, 6, 8, and 12 weeks, with all protocol durations exceeding 4 weeks. Exercises were typically repeated 30 or more times per session, while their frequency was outlined as two, three, or five times per week. Exercises were completed solely in clinic [2, 3, 37, 38] or with some form of clinical guidance from a speech-language pathologist or an occupational therapist along with self-directed home training [1, 29, 39] (see Table 4). Additionally, intervention for the treatment groups was not always limited to tongue interventions and included conventional dysphagia therapy techniques [37-39] such as effortful swallowing, thermal tactile stimulation, facial massage, and compensatory maneuvers or range of motion exercises.

**Table 4:** Training protocols

Study	Exercise	Device or tool	Repetition	Frequency (days/week)	Duration (weeks)	Guidance
Cho et al. [39]	Press tongue strongly against hard palate	None	30 times	5	4	Education provided by OT on day 1 then supervised by caregiver
Kim et al. [37]	TPRT	IOPI	30 times	5	4	Two experienced OTs (no home practice)
Park et al. [38]	Tongue muscle strength training (ant and post)	IOPI	10 × 5 (total = 50 times)	5	6	Two experienced OTs (no home practice)
Robbins et al. [1]	Isometric lingual exercise program (anterior and posterior)	IOPI	10 × 3 each location (total = 60 times)	3	8	SLP contact by telephone or in person during the initial week then every 2 weeks thereafter
Robbins et al. [28]	Isometric lingual exercise program (anterior)	IOPI	30 × 3 (total = 90 times)	3	8	Contact with SLP at baseline, weeks 2, 4, and 6 paired with home practice (daily log)
Steele et al. [2]	Maximum isometric tongue pressures (anterior and posterior) + amplitude accuracy	IOPI	60 times	2	11–12	Direct supervision by a licensed SLP in clinic (no home practice)
Steele et al. [3]	TPSAT –– TPPT	IOPI	60 times	2–3	8–12	Direct supervision by a licensed SLP in clinic (no home practice)

*OT* Occupational Therapist, *SLP *Speech-Language Pathologist, *TPRT *Tongue to palate resistance training (anterior and posterior), *IOPI* Iowa Oral Performance Instrument, *TPSAT* tongue pressure strength and accuracy training, *TPPT* tongue pressure profile training

#### Question 2: Swallowing function

##### VFSS Protocols

The seven studies included for review employed a broad range of VFSS protocols to assess swallow function post-treatment (see Table 5). None of the included studies reported the frame rate at which their VFSS studies were captured and recorded. Frame rate has been noted to interfere with the integrity of VFSS analysis if below 15 frames per second, particularly with respect to identifying penetration-aspiration events [40].

**Table 5:** Videofluoroscopy protocols

Study	Thin	Thick	Puree	Semisolids	Solid	No. of trials	Frames (s)
Cho et al. [39]	NR	NR	NR	NR	NR	NR	NR
Kim et al. [37]	NR	NR	NR	NR	NR	NR	NR
Park et al. [38]	NR	NR	NR	NR	NR	NR	NR
Robbins et al. [1]	Varibar thin liquid	No	No	Varibar pudding	No	*Thin* 3× (3 ml spoon, 10 ml catheter syringe); 2× (effortful 3 ml spoon) *Semisolid*: 3×: 3 ml spoon	NR
Robbins et al. [28]	3:1 (water:liquid Polibar plus)	No	No	Varibar pudding	No	*Thin* 3× (3 ml spoon, 10 ml catheter syringe); 2×: (effortful 3 ml spoon) *Semisolid*: 3×: 3 ml spoon	NR
Steele et al. [2]	Thin solution of polibar and water (40% w/v)	No	EZ-HD barium powder with applesauce (40% w/v)	No	No	*Thin* 3×: 5 ml spoon *Puree* 3×: 5 ml spoon	NR
Steele et al. [3]	Bracco EZ-Paque powder barium mixed with water (20% w/v)	Nectar: Bracco EZ-Paque powdered barium, xanthan gum thickener, mixed with water (20% w/v)	No	No	No	*Thin* 3× teaspoon amount *Thick* 3× teaspoon amount	NR

*NR* not reported, *No*  stimulus not used, *w/v* weight to volume ratio, *x* repetitions

It is important to note that not all studies disclosed their VFSS protocols, which would hinder replication of results; however, those that reported their VFSS protocols included a thin barium stimulus to evaluate the effects of the intervention [1–3, 29]. Information about barium concentration and brand of barium used was reported in all studies reporting VFSS protocol. Other stimuli included in the VFSS protocols varied across studies: (1) “thickened” stimuli [3], “puree” stimuli [2], and semisolid stimuli [1, 29]. No study utilized a solid consistency as part of their VFSS protocol.

The range of bolus volumes included was:

- *Thin* teaspoon, 2.5 ml spoon, 3 ml spoon, 5 ml spoon, 10 ml catheter syringe, and consecutive swallow task (unreported volume);
- *Thick* teaspoon;
- *Puree* 5 ml spoon, 10 ml spoon, 15 ml spoon;
- *Semisolids* ½ wafer dipped in barium, and 3 ml spoon.

##### Swallowing Biomechanics

Each study along with its inclusion criteria, study design, and a list of the outcome measures collected is shown in Table 6. Change in swallowing physiology was reported as an outcome of interest in six of the studies included in this review. The most commonly collected outcome measures of swallowing physiology to determine changes pre- and post-treatment were temporal measurements. Temporal measures included: oral transit time, pharyngeal transit time, stage transition duration, oral transit duration, oral clearance duration, pharyngeal transit duration, pharyngeal clearance duration, pharyngeal response duration, duration of hyoid maximum elevation, duration of hyoid maximum anterior excursion, duration to upper esophageal sphincter (UES) opening, duration of UES opening, total swallowing duration. Of these studies, three used the Videofluoroscopic Dysphagia Scale (VDS) as the tool of measurement; these studies did not report scores per parameter but instead reported compiled scores out of 100 across all parameters [41]. The VDS tool characterizes swallowing impairment based on ordinal scales for 14 parameters related to the oral and pharyngeal stage of swallowing, including some physiological measures (e.g., trigger of pharyngeal swallow, laryngeal elevation, pharyngeal transit time), and appears to be popular in Korea.

**Table 6:** Swallowing measures

Study	Inclusion criteria	Study design	Outcome measures collected
Cho et al. [39]	(1) Oropharyngeal dysphagia confirmed by VFSS; (2) No significant cognitive difficulties (MMSE > 24); (3) Ability to actively cooperate	Prospective Cohort Intervention Study	VDS (oral phase; pharyngeal phase)
Kim et al. [37]	(1) Post-stroke oropharyngeal dysphagia confirmed by a VFSS; (2) Tongue muscle strength < 10 kPa; (3) MMSE score > 20; (4) Able to swallow voluntarily; (5) Cortex damage only;	Pre–post-treatment design	Tongue strength (anterior; posterior) Posterior tongue strength VDS (oral phase; pharyngeal phase) PAS
Park et al. [38]	(1) Dysphagia from a stroke that was confirmed by a VFSS; (2) Onset duration > 6 months, (3) MMSE score ≥ 24	RCT	VDS (oral phase; pharyngeal phase; total score) Tongue strength (anterior; posterior)
Robbins et al. [1]	(1) 45 years of age or older; (2) Had a history of ischemic stroke; (3) Showed reduced lingual pressures with either the anterior or posterior tongue defined as < 40 kPa); (4) Physician referral for a VFSS that confirmed the presence of aspiration, penetration, or oropharyngeal residue	Prospective Cohort Intervention Study	PAS Durational measures (oral transit duration; oral clearance duration; pharyngeal transit duration; pharyngeal clearance duration; pharyngeal response duration; duration of hyoid maximum elevation; duration of hyoid maximum anterior excursion; duration to UES opening; duration of UES opening; total swallowing duration) Residue (oral cavity; vallecula; posterior pharyngeal wall; pyriform sinus; UES) Swallowing pressures Maximum isometric pressures (anterior and posterior) MRI (total lingual volume)/SWAL-QOL/Dietary intake questionnaire
Robbins et al. [28]	(1) No history of swallowing problems or medical conditions that would affect oral motor performance, such as a history of acute or degenerative neurological condition or head/neck cancer	Prospective Cohort Intervention Study	PAS Durational measures (oral transit duration; stage transition duration; pharyngeal transit duration; pharyngeal response duration; duration of hyoid maximum elevation; duration to UES opening; duration of UES opening; total swallowing duration) Residue (oral cavity; vallecula; posterior pharyngeal wall; pyriform sinus; UES) Swallowing pressures Maximum isometric pressures(anterior and posterior) MRI (total lingual volume)
Steele et al. [2]	(1) Dysphagia secondary to acquired brain injury following a motor vehicle accident; (2) Impaired swallowing safety, i.e., scores less than or equal to 3 on the PAS with thin liquids; (3) Post-swallow residues in the valleculae or pyriform sinuses with either thin and/or spoon-thick liquids measured using a 4-point ordinal scale	Case series	Isometric pressures (anterior; posterior) Saliva swallow pressures PAS Residue (vallecular; pyriform sinus)
Steele et al. [3]	(1) History of recent stroke (4–20 weeks prior to enrollment); (2) One posterior maximum isometric pressure < 40 kPa; stage transition duration > 350 ms on at least one thin liquid barium swallow during intake VFSS; (3) Able to understand English; (4) Able to follow directions; (5) Able to tolerate oral trials under the supervision of a therapist	RCT	Posterior maximum isometric tongue-palate pressures Stage transition duration on thin liquid swallows PAS Residue (vallecular)

*VFSS* Videofluroscopic Swallowing Study, *VDS* Videofluoroscopic Dysphagia Scale, *MMSE* Mini-Mental Status Examination score, *PAS *Penetration-Aspiration Scale, *RCT* Randomized Control Trial, *UES* Upper Esophageal Sphincter, *MRI* Magnetic Resonance Imagine, *SWAL-QOL* Quality of Life in Swallowing Disorders Questionnaire

##### Swallowing Safety

All studies reported swallowing safety as an outcome of interest following lingual resistance training. The Penetration-Aspiration scale (PAS) [42] is an 8-point scale grading the depth of penetration and aspiration of the bolus into the laryngeal vestibule along with subject response. The PAS was the primary tool used to quantify swallowing safety [1–3, 29], while the aspiration parameter on the VDS was used by the remaining studies [37–39] to assign scores related to presence of laryngeal vestibule invasion, supraglottic penetration, and subglottic aspiration. One article [37] reported swallowing safety using both the VDS and PAS scales. Reports of penetration-aspiration were provided using either ordinal scales or percentage estimates of the amount of the bolus aspirated in these studies.

##### Swallowing Efficiency

Swallowing efficiency measures were reported in all included studies as an outcome of interest following intervention. Areas for residue measurement were the vallecula [1–3, 29, 37-39], oral cavity [1, 29, 37–39], posterior pharyngeal wall [1, 29, 37–39], pyriform sinus [1, 2, 29, 37–39], and (UES) [1, 29]. Residue was quantified using a number of ordinal scales, including the following:

- 4-point ordinal system [2] (0 = none, 1 = less than 25% full, 2 = 25–50% full, 3 = more than 50% full) described by Eisenhuber et al. [43]
- Normalized Residue Rating Scale (NRRS) [44], which uses pixel-based measurements of space and residue normalized to an anatomical scaling factor to correct for differences in height using the cervical spine [3].
- 3-point system [1, 29] (0 = no residue; 1 = coating of barium residue; 2 = pooling of barium).
- 4 coded values on a nominal scalar mapped to scores on the VDS (0 = None; 4.5 =  < 10%; 9 = 10–50%; and 13.5 =  > 50%) [41].

#### Question 3: Other Measures

Other measures used to determine the effects of lingual resistance training included magnetic resonance imaging (MRI) to evaluate the total lingual volume [1, 29], a dietary intake questionnaire [1], and the Quality of Life in Swallowing Disorders Questionnaire (SWAL-QOL) [45] to quantify changes in swallowing related quality of life [1].

#### Question 4: Lingual Resistance Training Intervention Outcome

##### Tongue Pressure Generation

*Isometric Tongue Pressures:* Isometric tongue-palate pressures post-treatment was measured as an outcome of interest in a total of six studies, at either the anterior region, posterior region, or both regions. In all four studies assessing outcomes in the *anterior region* [1, 2, 29, 38], improvement was found and a statistically significant increase in pressures was reported in three studies. The fourth of these studies [2] used single subject methods for reporting results, and reported that 5/6 participants achieved improvement defined as three successive sessions in which pressures fell above a Cohen’s *d* effect size threshold of 0.6 versus baseline. For the *posterior region*, four studies reported significantly increased posterior isometric pressures between baseline and post-treatment measures [1, 3, 29, 38] and a fifth [2] reported improvements in all participants using the Cohen’s *d* effect size criterion. One study reported statistically significant increases in peak isometric pressures, however, no information was provided regarding the placement of the air filled bulb used to measure these pressures [29].

*Swallowing Pressures:* Tongue pressures collected using a three-bulb array attached along the midline of the hard palate were also reported in two studies [1, 29]. These pressures differ from isometric tongue pressures as they were collected during VFSS while patients were swallowing different bolus consistencies. A significant effect of bolus type was reported in one study, where maximum swallowing pressures increased significantly for all consistencies post-treatment except for the 3-ml-thin liquid task [29]. In the other study, significant increases in swallowing pressures were reported in at least one of three trials of 3 ml thin liquid, 10 ml thin liquid, and semisolid bolus conditions [1]. One other study collected tongue pressure measurements during swallowing tasks by utilizing saliva swallows. Half of their participants demonstrated increased saliva swallowing pressures beyond the effect size boundary for at least three consecutive sessions [2].

##### Swallowing Outcomes

*Temporal Measures*: No statistically significant changes in timing measures of swallowing were found in two [3, 29] of the three studies that collected them. In one study, oral transit duration (defined as time from beginning of posterior bolus movement until arrival of bolus head at ramus of mandible) [1] significantly improved for the 3 ml liquid bolus conditions in one of three bolus trials. Similarly, a significant effect was found for pharyngeal response duration (defined as time from beginning of hyoid excursion until hyoid returns to rest) for the 3 ml liquid and 10 ml liquid bolus conditions, also observed on one of three bolus trials per consistency. No additional physiological measures collected showed statistically significant changes (e.g., duration of pharyngeal response, UES opening, time to UES opening, hyoid maximum elevation, and hyoid maximum anterior excursion).

*Swallowing safety:* Swallowing safety pre- and post-treatment on VFSS was measured using PAS in most studies, with mixed results. In two studies, no significant improvements (i.e. decreases) in PAS were found for thin [1, 3], nectar [3], or pudding consistencies [1]. Significantly decreased (i.e. improved) PAS values were reported in three studies, two of which provided information regarding bolus stimuli used to assess swallow safety. As the remaining studies used the VDS tool and did not dissociate scores related to the swallow safety parameter from other parameters when reporting results, overall effects of the intervention on swallowing safety alone could not be extracted.

*Swallowing Efficiency:* A statistically significant reduction in vallecular residue was noted in NRRS scores for thin liquid stimuli in one study [3], while no significant differences were found for nectar-thick stimuli or for any other bolus type in the remaining studies. All studies, except one [1], reported no significant decreases in either oral cavity or pyriform sinus residue. In this study by Robbins et al. [1], the authors concluded that mean oropharyngeal residue scale scores changed significantly for three bolus conditions (3 ml effortful, 3 ml liquid, and 10 ml liquid), however repeated measures were not accounted for.

*Other measurements:* Oral phase parameters (lip closure, bolus formation, mastication, apraxia, tongue to palate contact, premature bolus loss, and oral transit time) and pharyngeal phase parameters (trigger of pharyngeal swallow, vallecular residue, laryngeal elevation, pyriform sinus residue, coating on the pharyngeal wall, pharyngeal transit time, and aspiration) as captured on the VDS were reported to significantly improve in all three studies using this tool. Data relating to each specific parameter were not reported in any of these studies. Detailed information regarding all reported outcomes, including results for MRI measures, quality of life measures, and dietary intake questionnaires, are given in Table 7.

**Table 7:** Summary of outcome measures and results

Study	Measures	Results
Cho et al. [39]	VDS (oral phase; pharyngeal phase)	Significant improvement in oral phase components (*p* < 0.05) Significant improvement in the pharyngeal phase components (*p* < 0.05)
Kim et al. [37]	Anterior tongue strength	Baseline MIP = 32.67 kPa; post-treatment MIP: 41.89 kPa Significant increases for both experimental and control; Statistically significant difference between groups (*p* < 0.000)
	Posterior tongue strength	Baseline MIP = 28.06 kPa; post-treatment MIP: 39.11 kPa Significant increases for both experimental and control; Statistically significant difference between groups (*p* < 0.000)
	VDS (oral phase; pharyngeal phase)	Significant improvements in both oral and pharyngeal phase of VDS for experimental and control groups (*p* < 0.000), and also between groups (*p* < 0.000)
	PAS	Significant decrease in PAS for both groups (*p* < 0.000); No significant differences between groups (*p* = 0.0471)
Park et al. [38]	VDS (oral phase; pharyngeal phase; total score)	Statistically significant differences in both the oral (*p* < 0.01) pharyngeal (*p* < 0.05) stages, and the total score (*p* < 0.01) for the experimental group. No significant difference in VDS scores between the experimental and control groups after the intervention
	Anterior tongue strength	Baseline MIP = 18.93 kPa; post-treatment MIP: 20.73 kPa Significant improvements for the anterior region pre–post-intervention for the experimental group (*p* < 0.01); no statistically significant difference between the two groups
	Posterior tongue strength	Baseline MIP = 16.2 kPa; post-treatment MIP: 18.47 kPa Significant improvements for the posterior region pre–post-intervention for the experimental group (*p* < 0.01); no statistically significant difference in scores between the two groups
Robbins et al. [1]	PAS	Significantly reduced (increased safety) for the 3-ml thin liquid bolus condition at week 4 (*p* = 0.02) and week 8 (*p* = 0.005); 10-ml liquid bolus condition after at 8 weeks (week 4: *p* = 0.08; week 8: *p* = 0.003). A trend toward reduced airway invasion for the effortful swallowing condition was statistically significant after 4 weeks of exercise (week 4: *p* = 0.03, week 8: *p* = 0.07)
	Durational Measures (oral transit duration; oral clearance duration; pharyngeal transit duration; pharyngeal clearance duration; pharyngeal response duration; duration of hyoid maximum elevation; duration of hyoid maximum anterior excursion; duration to UES opening; duration of UES opening; total swallowing duration)	Significant decrease in the oral transit duration (time from beginning of posterior bolus movement until arrival of bolus head at ramus of mandible) for the 3-ml liquid bolus condition (*p* = 0.036) on 1 of 3 trials An increase in the pharyngeal response duration (time from beginning of hyoid excursion until hyoid returns to rest) for both the 3-ml liquid (*p* = 0.02) and the 10-ml liquid (*p* = 0.024) bolus conditions on 1 of 3 trials
	Residue (oral cavity; posterior pharyngeal wall; pyriform sinus; UES)	Significant reduction in overall residue for the 3-ml effortful swallow (*p* = 0.02), 10-ml (*p* = 0 .02), and 3-ml (*p* = 0.01) bolus conditions, with the most significant changes in pharyngeal residue (*p* = 0.03) Reduction of average residue in the oral cavity (*p* = 0.07) and cricopharyngeus (*p* = 0.09) at week 8. No significant changes in average residue in the piriform sinuses (*p* = 0.17) or vallecula (*p* = 0.14) were observed after 8 weeks of exercise
	Swallowing pressures	Significant increases on at least 1 of 3 trials for: 10 ml liquid (week 4 *p* = 0.04, week 8 *p* = 0.03), 3 ml liquid (week 4 *p* = 0.04, week 8 *p* = 0.004), Semisolid bolus conditions (week 4 *p* = 0.05, week 8 *p* = 0.02)
	Maximum isometric pressures (anterior and posterior)	Anterior location: Baseline MIP = 35.6 kPa; Post-treatment MIP: 51.8 kPa Posterior location: Baseline MIP = 30.2 kPa; Post-treatment MIP: 54.6 kPa Statistically significant increase in anterior (week 4 *p* < 0.001, week 8 *p* < 0.001) and posterior tongue sites (week 4 *p* = 0.01, week 8 *p* < 0.001)
	MRI (total lingual volume)	Increases lingual volume for two of three subjects (mean = 4.35%); A decline for one patient (6.5%)
	SWAL-QOL	Statistically significant (fatigue, *p* = 0.047; communication, *p* = 0.026; and mental health *p* = 0.022)
	Dietary intake questionnaire	Six subjects reported addition of difficult-to-swallow food items (nuts, popcorn, salad, and raw vegetables) to their diet
Robbins et al. [28]	PAS	No significant changes
	Durational Measures (oral transit duration; stage transition duration; pharyngeal transit duration; pharyngeal response duration; duration of hyoid maximum elevation; duration to UES opening; duration of UES opening; total swallowing duration)	No significant changes
	Residue (oral cavity; posterior pharyngeal wall; pyriform sinus; UES)	No significant changes
	Swallowing pressures	Significant based on bolus type (*p* = 0.47): 3 ml effortful swallow: statistically significantly increase (*p* = 0.001) 10 ml of thin liquid: statistically significantly increase (*p* = 0.04) 3 ml semisolid: statistically significantly increase (*p* = 0.01) 3 ml thin liquid: not significantly changed (*p* = 0.18) No significant changes in pressure rise rate overall or by bolus type
	Maximum isometric pressures (anterior)	Baseline MIP = 41 kPa; Post-treatment MIP: 49 kPa A significant increase in peak isometric pressure (week 4, *p* = 0.002; week 6, *p* = 0.001)
	MRI (total lingual volume)	Increased lingual volume (mean change = 5.1%)
Steele et al. [2]	Anterior isometric pressures	Increases (sustained above the Cohen’s *d* = 0.6 effect size boundary for at least three consecutive sessions) for participants 1, 2, 3, 4, 5
	Posterior isometric pressures	Increases (beyond the *d* = 0.6 effect size boundary over at least three consecutive treatment sessions) for all six participants; sustained gains not seen for participant 1
	Saliva swallow pressures	Increases (beyond the effect size boundary over at least three consecutive sessions) for participants 1, 2, and 6, but gains were not clearly sustained for participants 2 and 6 Participants 3, 4, and 5 failed to demonstrate any notable changes in saliva swallowing pressures
	PAS	Thin liquids: Improved swallowing safety (participants 1, 2, 4, 5, and 6); unchanged (participant 3) Spoon-thick liquids: post-treatment improvement of all those unsafe at baseline (participants 1, 2, 3, and 6);
	Vallecular residue	Thin liquids: remained unchanged (three participants); worsened (three participants) Spoon-thick liquids: remained unchanged (two participants); worsened (4 participants)
	Pyriform sinus residue	Thin liquids: improved (one participants); remained unchanged (2 participants) and worsened (3 participants) Spoon-thick liquids: improved (2 participants) remained unchanged (2 participants) and worsened (2 participants)
Steele et al. [3]	Maximum isometric pressures (posterior)	Pooled MIPs across groups: Baseline MIP = 21 kPa; Post-treatment MIP: 41 kPa Significant increases post-treatment for the entire to mean values of 41 kPa (*p* < 0.001; Cohen’s *d* = 1.15) An significant main effect of protocol (*p* = 0.08; Cohen’s *d* = 1.64): lower pressures for TPPT participants Significant protocol X age interaction (patients over 80 in the TPPT group had the lowest tongue pressures) No significant differences in the magnitude of change between treatment groups and no protocol X age-group interactions
	Stage transition duration	Thin liquids swallows: No statistically significant change (*p* = 0.13)
	PAS	Thin & Nectar thickened liquids: There were no significant differences for either group, or across the entire pooled sample
	Residue (vallecular)	Thin liquid: Statistically significant reduction in NRRSv scores (p = 0.05; Cohen’s *d* = 0.58). There were no significant differences between treatment groups Nectar-thick liquids: Non-significant reduction but medium effect size (Cohen’s *d* = 0.54)

*VDS* Videofluoroscopic Dysphagia Scale, *MIP* maximum isometric pressure; *PAS* Penetration-Aspiration Scale, *RCT* Randomized Control Trial, *UES* Upper Esophageal Sphincter, *MRI* Magnetic Resonance Imagine, *SWAL-QOL* Quality of Life in Swallowing Disorders Questionnaire

## Discussion

### Risk of Bias

This review systematically examined the strength and quality of evidence for using lingual resistance training as an intervention to impact swallow function as measured using VFSS. The seven studies selected for review had mixed quality with four rated as “poor” on the risk of bias tools selected. Of note, performance bias was common as either blinding of participants and personnel during treatment or the blinding of individuals rating the VFSS to participants and time-point relative to intervention was not reported in any of the selected studies. Additionally, performance bias was rated as high on studies that did not appropriately handle the statistical analysis of a categorical (PAS) or ordinal outcome scale (VDS). Selection bias was also found in five studies as convenience sampling was mainly used with no randomization or concealment to treatment conditions. Furthermore, small sample size was identified as a limitation in 50% [1, 2, 29, 39] of included studies. This increases the risk of bias as it undermines the reliability of the results leading to lack of confidence that any statistically significant effect reflects a true effect at the population level.

Another common reason for lower quality ratings were concerns about the validity and reliability of the outcome measures used. In a recent study by Swan et al. [46] using the COnsensus-based Standards for the selection of health Measurement Instruments (COSMIN) process [47] to evaluate the psychometric quality of swallowing assessment tools, the VDS was found to have limited reliability, content validity, and indeterminate hypothesis testing (or item construct validity), while the PAS revealed conflicting findings in terms of reliability and intermediate content validity and hypothesis testing. Additionally, the lack of clarity with regards to intervention descriptions, delivery, and protocol adherence impacted quality assessment [1, 29, 37, 39]. The large heterogeneity in the patient populations, protocols used for training, and outcome measurement presented barriers for completing quantitative analyses on the data extracted.

### Patient Characteristics and Outcome Measures

This review identified mixed evidence that tongue intervention specifically impacts swallowing safety or efficiency in isolation; however, improved swallowing function (either safety and/or efficiency) was reported in six of seven studies reviewed [1–3, 37–39]. The only study that did not find significant improvement in swallowing function was one that recruited healthy older adults [29]. These results are not surprising given that the participants recruited did not present with swallowing impairments in the first place. Additionally, swallowing pressures, anterior, and posterior tongue strength were reported to significantly improve from baseline in all studies utilizing the IOPI as a measurement and training tool, even for the healthy older adult population; however, this did not have a direct relationship with either safety or efficiency changes.

Although positive evidence was found for the impact of lingual resistance training on swallow function, this is confounded by the heterogeneity of patient populations, training protocols, swallow function measurement, and other outcome measures seen across the selected studies. While the majority of patients who underwent lingual resistance training interventions in the included studies had a primary etiology of stroke, there was variation in the type of stroke (ischemic, hemorrhagic) and time post-onset of stroke (4 weeks– > 48 months). These differences in the studies recruiting stroke patients and the heterogeneity of patient population in other studies threatens the assumption that participants had similar swallowing impairment profiles to begin with, and may explain the variations seen in swallowing outcomes. Additionally, the observed variation in swallowing improvement may be attributable to differences in intervention protocols utilized, including training frequency (2–5 weeks), duration (4–12 weeks) and task repetition (30–90 times).

### Limitations

Although this review followed the PRISMA guidelines, it is not without its limitations. Firstly, a choice was made to exclude unpublished and grey literature from our literature search, which may explain the limited number of studies included in the review. Furthermore, studies were only included if VFSS measures were taken pre- and post-intervention, which resulted in the exclusion of studies which utilized only post-treatment VFSS or other instrumental assessments (e.g., fiberoptic endoscopic evaluation of swallowing) to determine effects of this type of intervention. The reason behind this was that we hoped to perform a meta-analysis to reach stronger conclusions regarding use of this intervention for swallowing, using quantitative analyses on extracted VFSS measures. Quantitative analyses were not possible for many reasons, particularly poor reporting of VFSS frame rate and stimuli (bolus consistencies and volumes) used during assessments. Finally, as limited translational resources were available, only English studies were included in this review. Despite this limitation, only one non-English study was excluded at the abstract screening stage and no studies were excluded at the full-text screening stage.

## Conclusions

Overall, this systematic review described the effects of lingual resistance training interventions on swallowing function. Consistent with previous reviews [4], positive evidence was found in terms of impact of these interventions on tongue pressures, along with mixed results for swallowing safety and efficiency. It is important to note that variability in the methodology with this intervention did not allow for quantitative meta-analysis or definitive conclusions. A lack of standardization in methods for VFSS measurement of outcomes across studies was found to be a particular barrier to data synthesis and meta-analysis. Controlled observational studies with larger sample sizes are still needed to provide clinical rationale for use of lingual resistance training in different clinical populations with dysphagia. Future investigations should focus on conducting instrumental evaluations and robust analyses using psychometrically sound instruments following lingual resistance training to provide stronger evidence of the efficacy of such training for improved swallow function.

## Electronic supplementary material

Below is the link to the electronic supplementary material.

Supplementary file1 (DOCX 13 kb)

Supplementary file2 (DOCX 13 kb)
