# Supplementary material for: The Effect of Lingual Resistance Training Interventions on Adult Swallow Function: A Systematic Review
Source: Dysphagia. 2019 Oct 14;35(5):745–61. doi: 10.1007/s00455-019-10066-1 (PMC7522100; doi:10.1007/s00455-019-10066-1)
Supplement: Supplementary file 1 — Supplementary file1 (DOCX 13 kb) [file 455_2019_10066_MOESM1_ESM.docx]

**Appendix A.** Search Strategy implemented for Ovid MEDLINE(R) Epub Ahead of Print, In-Process & Other Non-Indexed Citations, Ovid MEDLINE(R) Daily and Ovid MEDLINE(R) <1946 to June 11, 2018>

Search Strategy:

--------------------------------------------------------------------------------

1 exp Tongue/ (19926)

2 tongue?.tw,kf. (38515)

3 lingua*.tw,kf. (16844)

4 ((tongue? or lingua*) adj3 (strength* or pressur*)).tw,kf. (527)

5 or/1-4 (58952)

6 Deglutition/ (8845)

7 Deglutition Disorders/ (18245)

8 exp Larynx/ (37525)

9 Pharynx/ (18012)

10 Pharyngeal Muscles/ (1549)

11 Esophageal Sphincter, Upper/ (366)

12 swallow*.tw,kf. (26273)

13 deglut*.tw,kf. (4149)

14 Dysphagi*.tw,kf. (24500)

15 (pharynx or Pharyngeal).tw,kf. (30269)

16 (Larynx or laryngeal).tw,kf. (61687)

17 Hyolaryngeal.tw,kf. (84)

18 Cricopharyngeal.tw,kf. (972)

19 upper esophageal sphincter.tw,kf. (1047)

20 airway clos*.tw,kf. (556)

21 Sarcopenia/ (2603)

22 sarcopenia?.tw,kf. (5330)

23 Lateral Medullary Syndrome/ (682)

24 ((lateral or dorsolateral) adj2 medullary syndrome?).tw,kf. (182)

25 (wallenberg* adj2 syndrome?).tw,kf. (459)

26 (fluoro* or cinefluoro* or videofluoro*).tw,kf. (194364)

27 (modified adj2 barium).tw,kf. (332)

28 or/6-27 (355279)

29 5 and 28 (7767)

30 exp Exercise/ (166772)

31 Exercise Movement Techniques/ (593)

32 exp Exercise Therapy/ (42875)

33 exercis*.tw,kf. (264288)

34 (train or training).tw,kf. (364517)

35 protocol*.tw,kf. (370020)

36 rh.fs. (184311)

37 rehabilitation/ or "rehabilitation of speech and language disorders"/ (17638)

38 Speech-Language Pathology/ (2669)

39 or/30-38 (1169514)

40 29 and 39 (595)

41 ((tongue? or lingua*) adj3 (intervention? or prospective or treatment? or rehab*)).tw,kf. (768)

42 28 and 41 (118)

43 40 or 42 (690)

44 limit 43 to english language (599)

45 44 not (exp animals/ not exp humans/) (575)

46 45 not (exp children/ not exp adults/) (531)

***************************
