# Supplementary material for: The Effect of Lingual Resistance Training Interventions on Adult Swallow Function: A Systematic Review
Source: Dysphagia. 2019 Oct 14;35(5):745–61. doi: 10.1007/s00455-019-10066-1 (PMC7522100; doi:10.1007/s00455-019-10066-1)
Supplement: Supplementary file 2 — Supplementary file2 (DOCX 13 kb) [file 455_2019_10066_MOESM2_ESM.docx]

**Appendix B**: Data Extraction Sheet

| **Study** | | | | **Patient Characteristics** | | | | | | |
| --- | --- | --- | --- | --- | --- | --- | --- | --- | --- | --- |
| RefID # | Author | Year | Title | Study Design | Patient N (M,F) | Matched Control N (M,F) | Etiology | Age (Mean); | Control Age (Mean) | Inclusion/Exclusion Criteria |
|  |  |  |  |  |  |  |  |  |  |  |
|  |  |  |  |  |  |  |  |  |  |  |
|  |  |  |  |  |  |  |  |  |  |  |

| **Study** | | | | **Intervention Details** | | | | | |
| --- | --- | --- | --- | --- | --- | --- | --- | --- | --- |
| RefID # | Author | Year | Title | Protocol / Instructions | Device Used | Repetitions | Frequency (days/week) | Duration (weeks) | Guidance |
|  |  |  |  |  |  |  |  |  |  |
|  |  |  |  |  |  |  |  |  |  |
|  |  |  |  |  |  |  |  |  |  |

| **Study** | | | | **Videofluoroscopy Protocols** | | | | | | |
| --- | --- | --- | --- | --- | --- | --- | --- | --- | --- | --- |
| RefID # | Author | Year | Title | Thin | Thick | Puree | Semi-solids | Solids | # of trials | Frames/sec |
|  |  |  |  |  |  |  |  |  |  |  |
|  |  |  |  |  |  |  |  |  |  |  |
|  |  |  |  |  |  |  |  |  |  |  |

| **Study** | | | | **Intervention Outcomes** | |
| --- | --- | --- | --- | --- | --- |
| RefID # | Author | Year | Title | Outcome measure collected (Safety, Efficiency, Other visuo-perceptual or temporal swallowing parameters) | Statistical Results |
|  |  |  |  |  |  |
|  |  |  |  |  |  |
|  |  |  |  |  |  |
